# Biological activity of *Trachystemon orientalis* extracts against *Sitophilus oryzae* and *Oryzaephilus surinamensis*

**DOI:** 10.1038/s41598-026-47834-7

**Published:** 2026-04-11

**Authors:** Emre Şen, Hilal Susurluk

**Affiliations:** 1 Present Address: Research Centre for Fruit Growing, Institute of Horticultural Science, Hungarian University of Agriculture and Life Sciences, Elvira Major, Érd, 2030 Hungary; 2 Graduate School of Natural and Applied Sciences, Department of Plant Protection, Faculty of Agriculture, Bursa Uludag University, Bursa, Türkiye; 3 Department of Plant Protection, Faculty of Agriculture, Bursa Uludag University, Bursa, Türkiye

**Keywords:** Plant extracts, Boraginaceae, Stored product pests, Biological effects, Biochemistry, Plant sciences

## Abstract

Stored product pests, such as *Sitophilus oryzae* and *Oryzaephilus surinamensis*, pose significant threats to global grain storage. Plant extracts rich in phenolics offer environmentally friendly alternatives to synthetic pesticides for managing these pests. This study is the first to evaluate the biological activity of shade-dried (GS) and oven-dried (ES) water extracts of *Trachystemon orientalis* against *S. oryzae* and *O. surinamensis*. Phytochemical analysis revealed that the GS extract exhibited higher total phenolic content and antioxidant capacity compared to the ES extract. Specifically, the GS extract contained higher concentrations of abscisic acid (3346.76 mg/10^6^ g dried matter (dm)) and caffeic acid (1666.17 mg/10^6^ g dm), whereas the ES extract was richer in *o*- and *p*-salicylic acid, *p*-coumaric acid, and rosmarinic acid. Orientation assays conducted at four concentrations (62.5–500 ppm) revealed time- and concentration-dependent changes in the distribution of adults between treated and control compartments. After 120 h, the presence of *O. surinamensis* at the GS 500 ppm site decreased to 44% (G = 36, *p* ≤ 0.05), while *S. oryzae* presence dropped to 33% (G = 31.4, *p* ≤ 0.05). Furthermore, higher concentrations of the GS extract significantly reduced F1 adult emergence for both pests (*p* < 0.05, Two-Way ANOVA). These findings suggest that *T. orientalis* extracts, particularly GS, represent promising environmentally friendly alternatives to chemical pesticides for the management of stored product pests. The efficacy of these extracts is closely associated with their higher phenolic content and specific bioactive compounds.

**Supplementary Information:**

The online version contains supplementary material available at 10.1038/s41598-026-47834-7.

## Introduction

Cereals, one of the primary food sources used in human nutrition, hold a significant place worldwide, not only because of their role in human diet but also due to their substantial contribution to the global agricultural economy. Storage periods of cereals vary depending on the intended use and are realized as short or long-term storage. During this storage, products are exposed to damage by various factors, especially due to inappropriate storage conditions. Without modern storage techniques for cereal crops, damage from insects can reach as high as 20% or more, according to reports^1^.

*Sitophilus oryzae* (Linnaeus, 1763) (Col: Curculionidae) is one of the stored product pests causing significant losses, especially in tropical and subtropical regions. In addition to product losses by feeding on stored cereals and cereal products, they also cause a decrease in product quality, loss of bread-making properties, and inability to use the product economically with the waste and feces they leave^2^. The most commonly used method for the control of this pest is chemical control, and empty warehouse spraying with long-lasting active substances is one of the most frequently used methods. In addition to residual surface treatments, fumigation with phosphine or other gaseous insecticides has long been considered a primary strategy in the management of stored-product pests worldwide^3,4^. Although physico-mechanical and cultural control methods are used to control this pest, these methods are sometimes insufficient to achieve the desired success. Chemicals used in the control of this pest have significant negative effects on human health and the environment. In addition to these adverse effects, the development of resistance in pests complicates the control of pests and diseases that cause major economic losses and leads to a rapid increase in product losses^5,6^.

The saw-toothed grain beetle, *Oryzaephilus surinamensis* (Linnaeus, 1758) (Col: Silvanidae) is also an important stored product pest. The pests are found in stored cereals, rice, flour and its products, dried fruit, tobacco, and vegetables, but can not feed on undamaged seeds (secondary pests) feed intensively on infected crops and cause losses. The pest causes quality losses in the product due to its shirt residues, faeces, and web substances they secrete. In addition, intense contamination causes the temperature in the warehouse to rise, leading to moulding, browning, and stinking of the product^7^. If chemical control is not carried out regularly, it causes significant quantity losses in stored grain^8^.

In addition to fumigation practices, residual surface treatments using contact insecticides management with the stored product pests. Although pyrethroids such as deltamethrin, beta-cyfluthrin, and alpha-cypermethrin are widely applied as grain protectants against *S. oryzae*, their residual efficacy depends on dose and exposure time, and suboptimal concentrations may allow progeny development^9^. Although residual insecticides such as malathion, deltamethrin, and lambda-cyhalothrin are widely used in storage facilities, resistance development and variable surface efficacy necessitate alternative strategies, and information on fumigant effectiveness against phosphine-resistant *S. oryzae* populations remains limited^10^. Increased reliance on organophosphates, pyrethroids, and spinosyns for the control of *S. oryzae* has led to widespread resistance, highlighting the urgent need for effective resistance management strategies^11^. Beyond direct mortality, pyrethroids and neonicotinoids can induce significant sublethal behavioral alterations in *O. surinamensis*, suggesting that contact insecticides may influence warehouse pest management through both toxic and behavioral effects^12^. Due to the reduced efficacy of conventional insecticides, alternative contact insecticides have been increasingly evaluated against *O. surinamensis*, with recent studies indicating that compounds such as lambda-cyhalothrin and spinetoram exhibit substantial toxicity across multiple life stages of this economically important storage pest^13^. Collectively, these limitations emphasize the urgency of developing more effective and environmentally compatible management tools.

Although synthetic residual insecticides and fumigants continue to play a central role in stored-product protection, their long-term sustainability is increasingly questioned due to several limitations, including resistance development, detectable pesticide residues in stored commodities, environmental contamination, non-target toxicity, and potential human health risks. These concerns have led to stricter regulatory restrictions and highlight the importance of integrated pest management and diversified control options^4,14–18^.

In the world, alternative control methods that do not have negative effects on the environment or have limited negative effects, do not cause residual problems, and can be easily applied are being investigated in the control of agricultural pests. Among these studies, especially studies on plant-based insecticides have gained importance, and the number of studies in this field is increasing day by day^19–21^. As a result of intensive studies carried out especially in the last 20 years, extracts obtained from various plants are used effectively in the control of many pests from various orders either as they are or purified^21–27^.

While botanical pesticides such as neem-based formulations (azadirachtin), pyrethrum extracts, and clove or cinnamon oil products have demonstrated efficacy against stored-product insects in laboratory studies, their commercial availability for industrial grain storage remains very limited^28,29^. Among these, only azadirachtin (from neem) is explicitly reported as a commercially available botanical insecticide for stored grains^30^. Despite their laboratory efficacy, major constraints include high volatility, rapid degradation, short residual persistence, and the large quantities required for effective treatment^31,32^. These factors have restricted the widespread adoption of botanical grain protectants in industrial storage systems.

The natural distribution of *Trachystemon orientalis* G. Don (Linnaeus, 1837) (Boraginales: Boraginaceae) is generally known from Eastern Bulgaria to the Western Caucasus. However, it has also been recorded in Wetter, North Rhine-Westphalia, Germany^33^, Ireland, and the United Kingdom^34^. It is a rhizomatous geophyte and grows primarily in the temperate biome. It has antipyretic, blood-purifying, diuretic, and emollient effects. Its flower buds and leaves can be consumed as vegetables in the human diet. It contains tannin, resin, mucilage, nitrate salts, saponins, and essential oils^35,36^. Herbicide, antioxidant, and antifungal properties of the plant have been concluded in previous studies, and it was observed that it contains flavonoids and phenolic compounds^37–39^. According to the results of some studies, *T. orientalis* was shown to be a good source of natural antioxidants due to its anthocyanin, phenolic, and flavonoid contents^40,41^.

There are several studies on the effects of some plant extracts from the Boraginaceae family against stored product pests. Researchers reported that extracts of the Indian heliotrope *Heliotropium indicum* L. (Boraginaceae: Heliotropium) caused a small amount of repellency on the cowpea weevil *Callosobruchus maculatus* (Fabricius 1775) (Coleoptera: Chrysomelidae)^42^. Crude and ethyl acetate extracts of *Moltkiopsis ciliata* (Forssk 1953) (Boraginaceae: I. M. Johnst.) were reported to be highly toxic on *S. oryzae*^43^. It was reported that *Heliotropium bacciferum* Forssk (Boraginales: Boraginaceae) extract had highly toxic effects on *Oryzaephilus surinamensis* (Linn.) (Coleoptera: Silvanidae) adults^44^. Researchers concluded that *Cordia sinensis* Lam. (Boraginaceae: Cordia) extracts are a potential source of green pesticide against *S. oryzae*^45^. In a study, it was reported that the dichloromethane extract of the whole plant of *Heliotropium strigosum* (Willd 1798) (Boraginaceae: Heliotropium) showed low contact toxicity effects against *S. oryzae* while it showed moderate toxic effects on *Rhyzopertha dominica* (Fabricius 1792) (Coleoptera: Bostrichidae)^46^. Aqueous and ethanol extracts of *Trichodesma indicum* L. (Boraginaceae: Trichodesma) showed low activity in the adults of *S. oryzae* in different studies, on the contrary, n-hexane extract was effective on adults^47,48^.

Antioxidant-rich plant extracts containing phenolic and polyphenolic compounds have been reported to influence behavioral responses of stored-product pests, including *S. oryzae* and *O. surinamensis*^49–52^. Antioxidant capacity, commonly measured through assays such as DPPH radical scavenging and reducing power tests, often reflects the abundance of redox-active phenolics. Many of these aromatic compounds are known to interact with insect chemosensory systems and may function as deterrents or repellents through olfactory or contact-mediated pathways^53^. These findings suggest that phytochemical structure and aromatic configuration, rather than antioxidant capacity per se, are likely to play a determining role in modulating insect orientation responses. The phytochemical composition and antioxidant properties of aqueous extracts of *T. orientalis* were comprehensively characterized in a previous study^41^. Drying conditions are known to influence the stability, transformation, and extractability of phenolic compounds. In our previous study^41^, shade- and oven-drying of *T. orientalis* resulted in distinct phytochemical profiles and differential biological activities. Oven-dried aqueous extracts exhibited strong lethal effects on *Tetranychus urticae* Koch (Acari: Tetranychidae), whereas shade-dried methanolic extracts were enriched in rosmarinic acid and showed pronounced repellent activity^41^. Multivariate analyses confirmed that drying method significantly structured phenolic differentiation. Therefore, both shade-dried and oven-dried materials were deliberately selected in the present study to assess how drying conditions affect crude extract composition and bioactivity.

The present work, building on these results, is devoted exclusively to evaluating the biological activity of the same extracts. This is the first study to evaluate the biological effects of *T. orientalis* extract against the major stored-product pests *S. oryzae* and *O. surinamensis*. This work aims to fill the existing knowledge gap, as no studies have so far investigated the activity of *T. orientalis* extracts or essential oils on other stored-product pests, thereby providing novel insights into the potential of this plant as a sustainable pest management option.

## Material and methods

### Stock culture of the insects

Cultures of *Sitophilus oryzae* and *Oryzaephilus surinamensis* were maintained in the Department of Plant Protection, Faculty of Agriculture, Bursa Uludag University, Türkiye, following the protocol described by Karakoç et al.^54^. The insects were reared in five-liter transparent plastic containers (26 × 24 × 8 cm) filled to one-third of their volume with sterilized wheat grains for *S.oryzae*. Cultures were kept under controlled environmental conditions at 27 ± 1 °C and 65 ± 5% relative humidity. Cracked wheat grains were used as the rearing substrate for *O. surinamensis*, as described by Hassan et al.^55^.

To obtain age-synchronized adults, approximately 200 mixed-sex individuals were introduced into rearing jars containing sterilized wheat and allowed to oviposit for 48 h. Following the oviposition period, all parental adults were removed by sieving to ensure cohort uniformity. The cultures were subsequently maintained under the same environmental conditions until adult emergence. Newly emerged adults were collected daily, transferred to clean jars containing untreated wheat, and the date of emergence was recorded.

Adults used in the bioassays were selected between 7 and 14 days after emergence to ensure physiological maturity and peak reproductive activity. Previous studies have demonstrated that adults within this age interval exhibit the highest fecundity and feeding performance, representing the most biologically active cohort^56^. Therefore, individuals younger than 7 days or older than 14 days were excluded to minimize variability associated with incomplete sexual maturation or age-related behavioral decline.

### Plant material and distribution

*Trachystemon orientalis* specimens were collected in May 2022 from the lower slopes surrounding Süle village in the İnegöl district of Bursa (Marmara Region, Türkiye). The collection site is located at approximately 40.0389° N and 29.4001° E, at an elevation of about 600 m, within an area exhibiting transitional climatic features between the Black Sea and Mediterranean zones.

No special permit was required for collection because *T. orientalis* naturally occurs in this region and is not an endemic or threatened species in Türkiye. Its broad natural distribution across Eastern Europe, the Balkans, and the Caucasus has been well documented^57–60^. Since the species is widespread and naturally occurring, collection in its wild habitat complies with institutional, national, and international regulations, including the IUCN Policy Statement on Research Involving Species at Risk of Extinction, and does not involve any species listed in the CITES Appendices.

A voucher specimen was deposited in the Bursa Uludag University Herbarium (BULU) under accession number BULU 47731, collected by Hilal Susurluk, and is publicly accessible for future reference.

### Extraction method and the total phenolic content, antioxidant capacity, and polyphenols of the extracts

#### Extraction method

The collected plant material was subjected to two different drying procedures prior to extraction. For shade drying, the material was spread in thin layers and kept at room temperature in a dark, well-ventilated environment for three weeks, whereas oven drying was performed at 65 °C for 48 h in a temperature-controlled oven. After drying, samples from each treatment were finely ground and used for extraction. All prepared extracts were stored at − 20 °C until they were used in subsequent chemical analyses and bioassays. For the preparation of the aqueous extract (10%), 50 g of powdered plant material was mixed with 500 mL of sterile distilled water adjusted to pH 6.5 and extracted for 24 h under continuous agitation using an orbital shaker (SK-O330-Pro, DLAB). The resulting suspension was first passed through Whatman No. 1 filter paper for coarse clarification and subsequently centrifuged at 5000 rpm for 10 min at 4 °C using a CF15RN centrifuge (Hitachi), with the supernatant collected after each run; this step was repeated three times. As residual fine particles were still present after repeated centrifugation, the extracts were additionally treated in an ultrasonic bath (Elma LC30H, Darmstadt, Germany) for 15 min at 5 °C, followed by high-speed centrifugation at 15,000 rpm for 10 min at 4 °C. The final supernatant was passed through a 0.45 μm membrane filter, as described by Onaran and Yılar^38^. The water extracts obtained from shade-dried and oven-dried plant material were designated as GS and ES, respectively, as previously reported by Susurluk et al.^41^.

#### Total phenolic content

Total phenolic content was measured using the Folin–Ciocalteu assay following the method of Velioglu et al.^61^. Quantification was performed using gallic acid calibration curves prepared over a concentration range of 10–600 ppm, and results were expressed as milligrams of gallic acid equivalents (GAE) per 100 g of dry matter (dm). The calibration curve for the aqueous extract showed high linearity (R^2^ = 0.9985). The total phenolic contents of the ES and GS extracts used in the present study were 25.46 and 38.04 mg GAE/100 g dm, respectively, as previously reported by Susurluk et al.^41^.

#### Antioxidant capacity

The determination was performed using the copper (II) ion-reducing antioxidant capacity (CUPRAC) assay, as described by Apak et al.^62^. Results were expressed as micromoles of Trolox equivalents (TE) per gram of dm. Quantification was based on calibration curves prepared with Trolox standards, applying linear regression analysis over a concentration range of 10–800 ppm, which showed excellent linearity for the aqueous extracts (R^2^ = 0.9993). The CUPRAC values of the ES and GS extracts were 5.18 and 240.30 µmol TE/g dm, respectively.

#### Polyphenols

Individual polyphenolic compounds were identified and quantified using an LC–MS/MS 8060 system (Shimadzu, Kyoto, Japan) following a modified protocol based on Akpinar Bayizit et al.^63^. Chromatographic separation was carried out on a C18 column (3 × 100 mm, 3 μm; GL Sciences, Tokyo, Japan) at a flow rate of 0.4 mL/min with an injection volume of 10 μL, while the column temperature was maintained at 40 °C. A gradient elution system consisting of formic acid/MQ water (1:1000, v/v) as eluent A and formic acid/acetonitrile (1:1000, v/v) as eluent B was used. Mass spectrometric detection was performed in both positive and negative electrospray ionization (ESI) modes under multiple reaction monitoring (MRM) conditions. The main operating parameters included a nebulizing gas flow rate of 3.0 L/min, a drying gas flow rate of 10.0 L/min, an interface voltage of 4.0 kV, a desolvation line temperature of 250 °C, an interface temperature of 300 °C, and a heat block temperature of 400 °C. Data acquisition and processing were conducted using LabSolution software (Shimadzu).

### Orientation assay

**Fig. 1:**
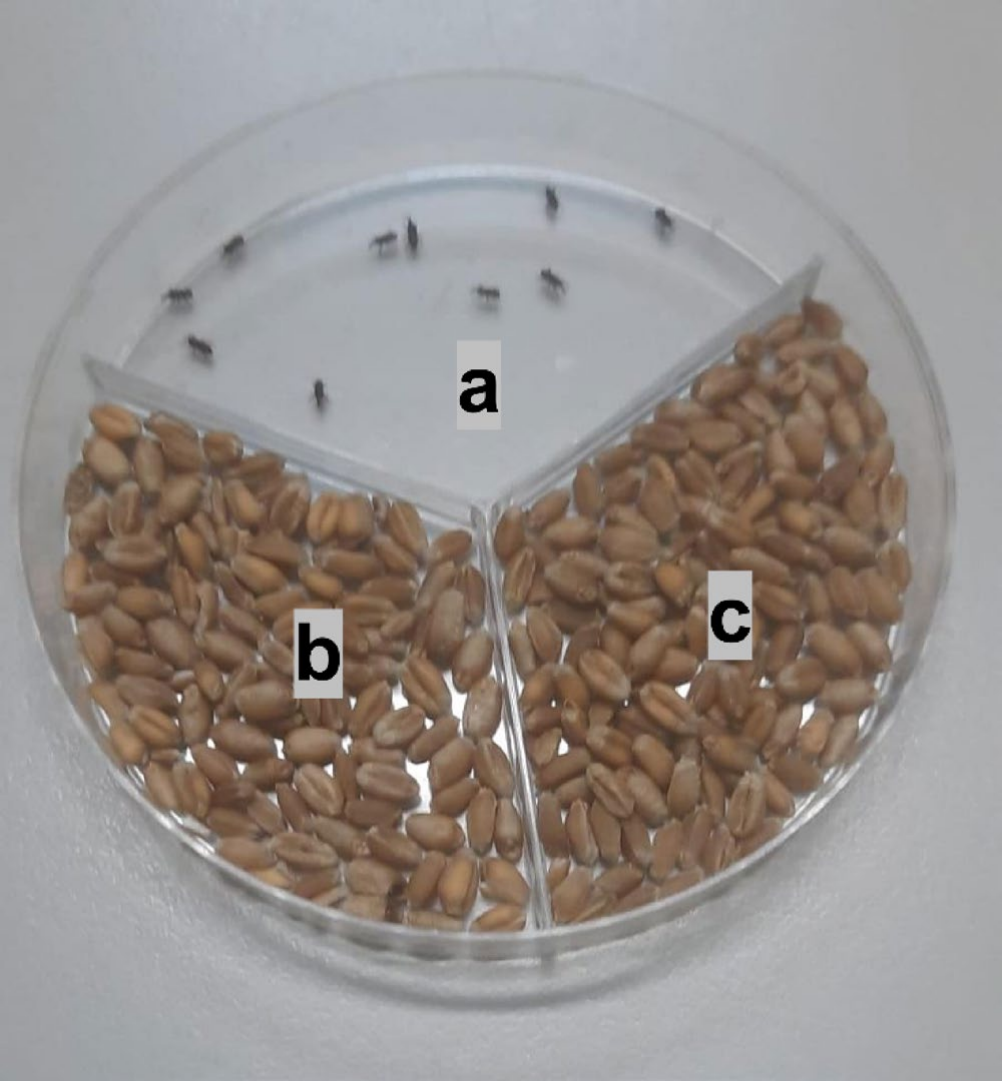
Experimental area. Three-compartment Petri dish showing (**a**) insect release area, (**b**) untreated area (control), and (**c**) treated wheat.

The methodology of Kestenholz^64^ was adopted in this study. Accordingly, 90 mm plastic Petri dishes (90 × 17 mm–gamma sterile) with 3 compartments were used in the experiment. Concentrations of 62.5, 125, 250, and 500 ppm of GS (10%) and ES (10%) extracts obtained by applying two different drying methods were used in the experiments. Five g each of wheat was weighed and placed in two separate compartments of the Petri dishes. From each prepared concentration, 300 µl were withdrawn with an automatic pipette and transferred onto 5 g of wheat. Another 300 µl of distilled water (control area) was added to the other 5 g of wheat and mixed thoroughly, and the wheat was allowed to absorb the extract and water for about half an hour. The third area was designed as a space for the release of insects (Fig. 1). 10 adults were released into the empty area, and the insects were transferred in closed Petri dishes to climate chambers at 27 ± 2 °C and 65% humidity. Petri dishes were wrapped with 2 layers of Parafilm to prevent insects from leaving the experimental area. In the experiment, insects moving toward three different compartments of Petri dishes were counted separately for each time point. The experiment was terminated after counting. Time periods were determined as 24, 48, 72, 96, and 120 h, and this orientation was calculated as a percentage. The study was designed as a randomized block design, with all groups and a control group in each block. The whole experiment was repeated on 3 different dates with four replicates. The orientation of *S. oryzae* and *O. surinamensis* adults towards treated, untreated (control), and indecisive areas was analyzed using a repeated-measures goodness-of-fit test (G-test) at a critical level of 5%^65^. The null hypothesis was that insects would show a 1:1 distribution between the treatment and control for each replicate. Orientation responses were interpreted based on the number and proportion of adult insects recorded in each compartment, using statistical comparisons between the treated and control compartments. A higher number of adults in the control compartment was interpreted as a repellent response, whereas a higher number in the treated compartment was interpreted as attraction. The absence of a significant difference between compartments was considered a neutral response. When a substantial proportion of adults remained in the neutral compartment without a clear preference for either side, the response was classified as indecisive.

### Effect on progeny

After 120 h of exposure in the orientation assay, all adults were removed from the Petri dishes. Wheat grains from each treatment and control replicate were transferred separately into glass jars (1.7 × 40 × 25 cm) for F1 assessment. A parallel control (four replicates of 10 untreated adults, allowed to mate for 120 h) was established. Following adult removal, F1 adult emergence was monitored weekly for 10 weeks, and progeny numbers were recorded. Data were analyzed in RStudio, with all experiments conducted in a climate chamber (27 ± 1 °C, 65% RH). Each treatment consisted of four independent replicates.

### Statistical analysis

The statistical analyses were performed in Rstudio (version 4.5.0) using the packages ggplot2, tidyr, dplyr, readr, writexl, broom, multcompView. Prior to analysis, data were tested for normality using the Shapiro–Wilk test and for homogeneity of variances using Levene’s test. Since the assumptions of normality and homoscedasticity were met, F1 adult emergence data were analyzed using two-way analysis of variance (ANOVA), with insect species and extract treatments as factors. When significant effects were detected, means were separated using Tukey’s HSD test (α = 0.05). Orientation data were analyzed using a goodness-of-fit G-test with Williams’ correction factor, testing the null hypothesis of a 1:1 distribution between treated and control compartments. All graphical representations were generated using ggplot2.

## Results

### Orientation assay

**Fig. 2:**
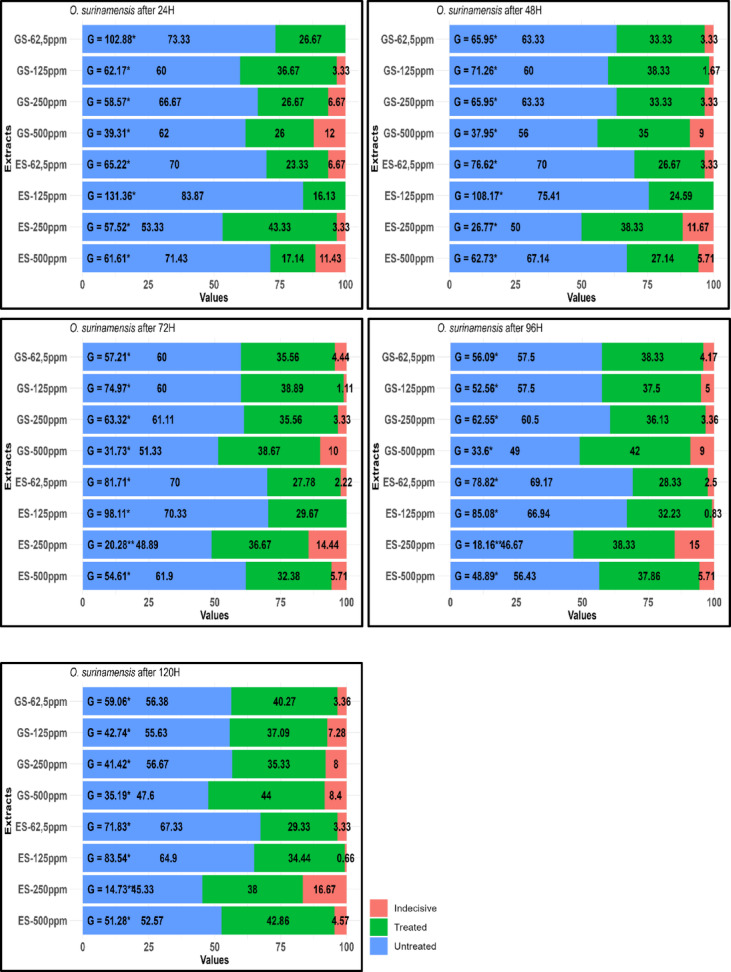
Orientation percentages (%) induced by GS and ES extracts from *Trachystemon orientalis* on *Oryzaephilus surinamensis*. GS represents water extracts from shade-dried plant material, while ES represents water extracts from oven-dried plant material. Significance levels for *p*-values are represented as *, **, corresponding to significant *p*-values of 0.05 and 0.01, respectively, based on the replicated goodness-of-fit test for G per replicate and GP.

Figure 2 summarizes the % orientation of *O. surinamensis* toward treated, untreated, and empty compartments of Petri dishes at various ES and GS extract concentrations (62.5, 125, 250, and 500 ppm) over specific periods. Statistical tests (G-values) and significance levels (*p*-values) were reported for each concentration. At the end of the 24-h experiment, the presence of *O. surinamensis* adults in the application area treated with the GS 62.5 ppm extract was 73.33% in the control group (G = 102.8, *p* ≤ 0.05). This ratio gradually decreased over time, reaching 56.38% at the end of the 120-h experiment (G = 59.06, *p* ≤ 0.05). For higher GS extract concentrations (125, 250, and 500 ppm), the number of adults in the control area declined as both concentration and exposure time increased. Notably, at the end of the 120 h trial, only 47.6% of adults were observed in the GS 500 ppm extract-treated area (G = 35.19, *p* ≤ 0.05). In contrast, the number of adults in the application area increased with higher GS extract concentrations after 120 h. Specifically, 40.27% of adults were found in the GS 62.5 ppm extract-treated area, while 44% adults were recorded in the GS 500 ppm extract-treated area (respectively, G = 59.06, *p* ≤ 0.05, G = 35.19, *p* ≤ 0.05) (Fig. 2).

Regarding the ES extract, the presence of insects in the control compartment was statistically significant at 70% for the ES 62.5 ppm concentration after 24 h. This percentage decreased over time, reaching 67.33% at the end of the 120-h experiment (respectively, G = 65.22, *p* ≤ 0.05, G = 71.83, *p* ≤ 0.05). In the case of other extracts and concentrations, the highest presence rate of *O. surinamensis* adults was observed in the control compartment at 24 h for the ES 125 ppm extract, where the rate was calculated to be 83.87%. By the end of the 120 h, this rate decreased to 64.90% in the control group (Fig. 2).

**Fig. 3:**
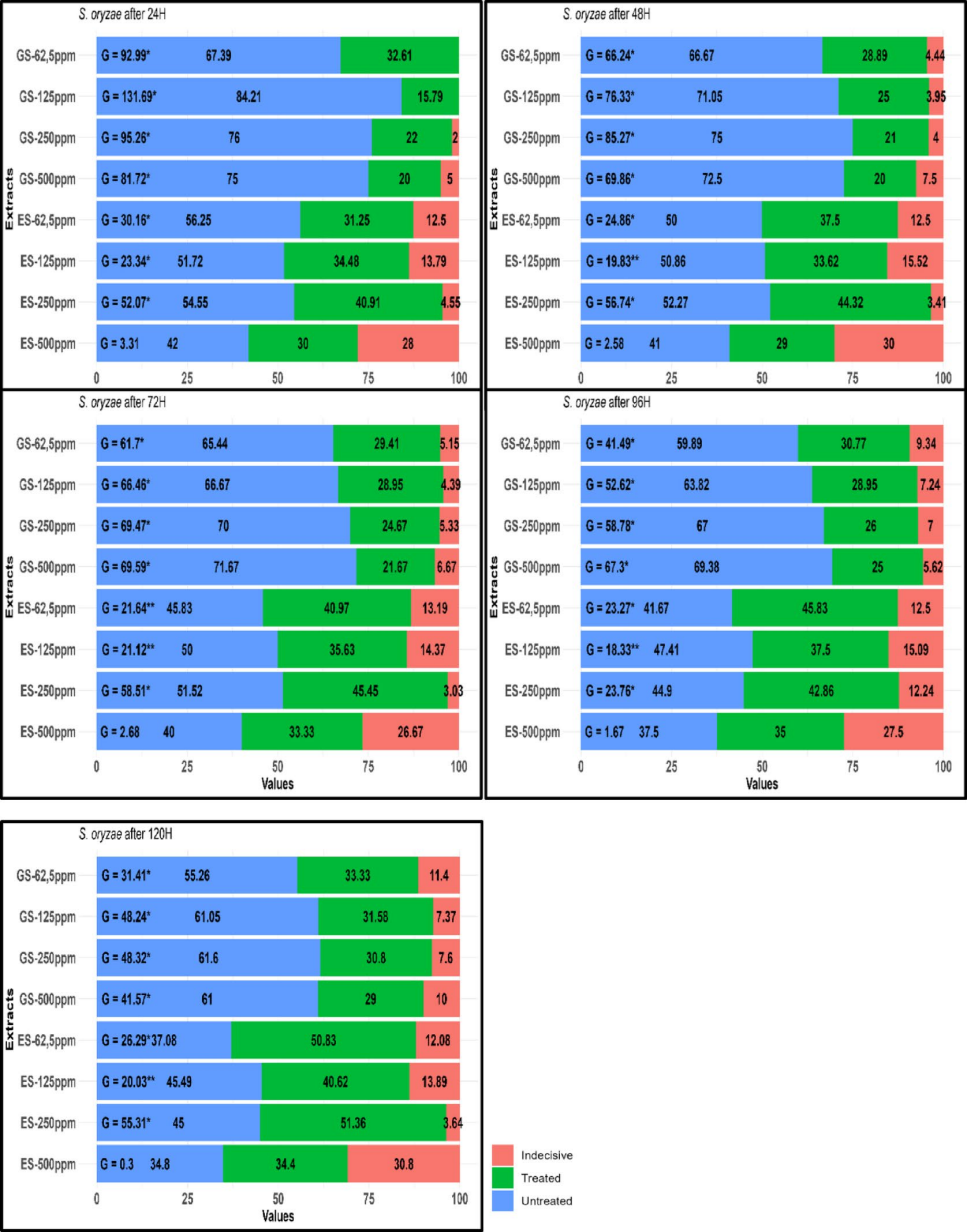
Orientation percentages (%) induced by GS and ES extracts from *Trachystemon orientalis* on *Sitophilus oryzae*. GS represents water extracts from shade-dried plant material, while ES represents water extracts from oven-dried plant material. Significance levels for *p*-values are represented as *, **, corresponding to significant *p*-values of 0.05 and 0.01, respectively, based on the replicated goodness-of-fit test for G per replicate and GP.

Figure 3 summarizes the % orientation of *S. oryzae* toward treated, untreated, and empty compartments of Petri dishes at various ES and GS extract concentrations (62.5, 125, 250, and 500 ppm) over specific periods. For *S. oryzae* adults, the presence rates in the control and application areas at the end of 24 h for the GS 62.5 ppm extract were calculated to be 67.39% and 32.61%, respectively (G = 92.99, *p* ≤ 0.05, G = 31.41, *p* ≤ 0.05) (Fig. 3). Conversely, the presence rate in the application area increased, reaching 33.33% after 120 h. Notably, the highest presence rate in the control group for the GS 125 ppm extract was observed at 84.21% after 24 h, which decreased to 61.05% at the end of 120 h. In the application area, the adult presence rate was 15.79% at 24 h and increased to 31.58% after 120 h. For the GS 250 ppm extract, the presence rates in the control group at the end of the 24 and 120 h were 76% and 61.6%, respectively, while in the application area, the rates were 22% at 24 h and 30.8% after at 120 h (respectively, G = 95.26, *p* ≤ 0.05, G = 48.31, *p* ≤ 0.05) (Fig. 3).

With the ES extract, the highest orientation behavior was recorded in the control group at 56.25% after 24 h for the ES 62.5 ppm concentration, which decreased to 37.08% at the end of the 120 h (respectively, G = 30.16, *p* ≤ 0.05, G = 26.28, *p* ≤ 0.05). However, as time progressed, the adult insects exhibited a notable attraction to the odors produced by the extracts. At the end of the 120 h, 50% proportion of adults were detected in the application area. In the other concentrations of this extract, the initial presence of adults in the control area was higher than average. Over time, a higher proportion of adults was recorded in the application area at later time points. However, as exposure time increased, a higher proportion of adults was recorded in the treated compartments. At the end of the 120 h, 40.63% of adults were detected in the application area (G = 20.03, *p* ≤ 0.0001). In the case of the ES 250 ppm extract, 54.55% of adults were observed in the control group at 24 h, while 51.36% of adults were found in the application area at 120 h (respectively, G = 52.07, *p* ≤ 0.05, G = 65.31, *p* ≤ 0.05). Similarly, for the ES 500 ppm extract, 42% of adults were recorded in the control group at 24 h, and 34.4% of adults were detected in the application area at the end of the 120 h. Statistically, the insects in the ES extracts were found in the application area at the following rates at the 120 h: 50.83%, 40.63%, 51.36%, and 34.4% for the ES 62.5 ppm, 125 ppm, 250 ppm, and 500 ppm extracts, respectively (Fig. 3).

### Effects of extracts on progeny of treated pests

**Fig. 4:**
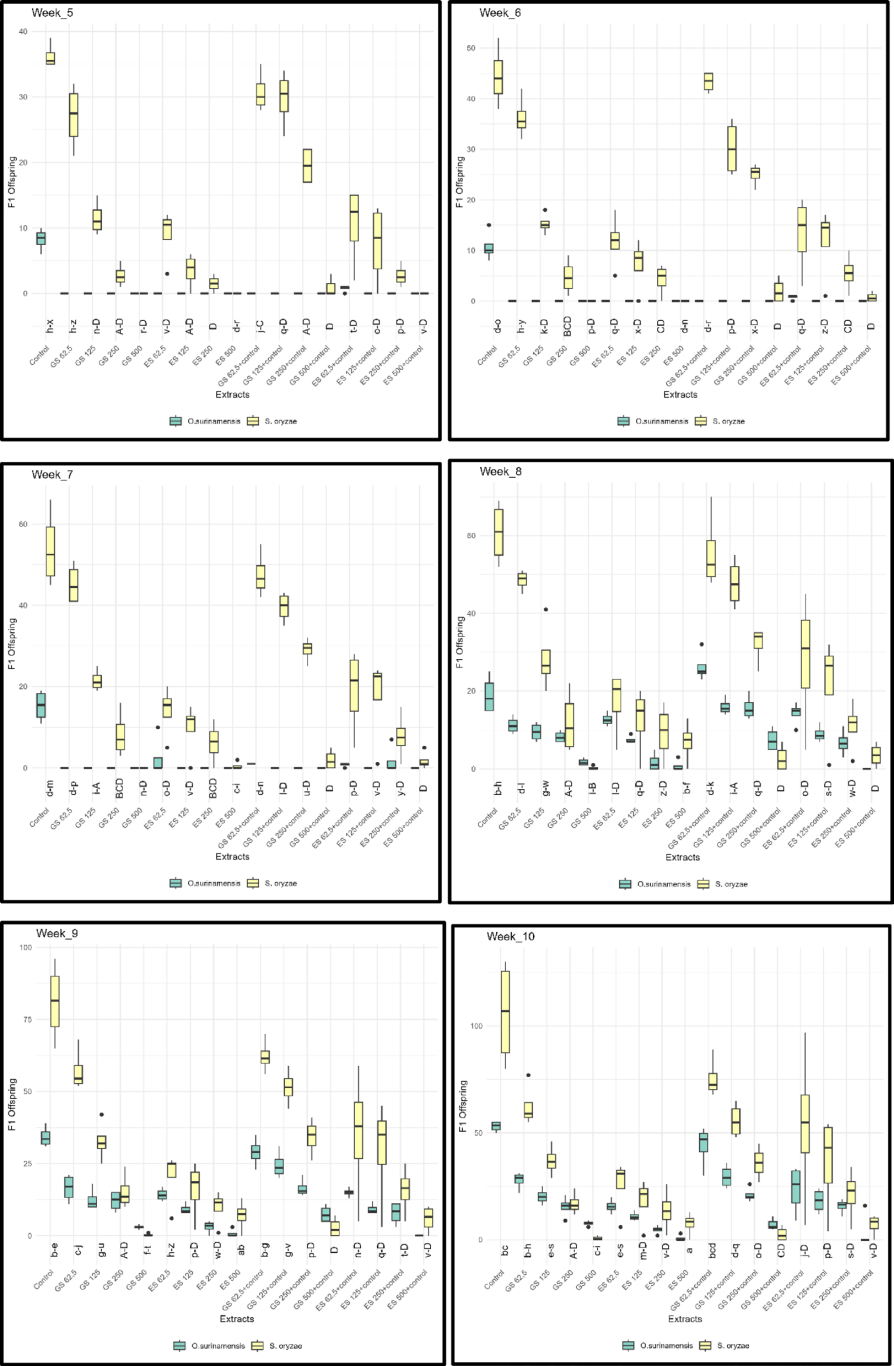
Effect of GS and ES extracts obtained from *Trachystemon orientalis* on the F1 generation of *Sitophilus oryzae* and *Oryzaephilus surinamensis*. GS represents water extracts from shade-dried plant material, while ES represents water extracts from oven-dried plant material.

Insect species, extract type, and the interaction of these two factors showed statistically significant effects on F1 offspring (*p* < 0.001). The effect of insect species was found to be quite strong and was calculated as F (1, 781) = 365.43, G = 43,21. Extract type also showed a significant effect on F1 offspring (F (16, 781) = 62.73, G = 118,69). The interaction between insect species and extract type was also found to be statistically significant (F (16, 781) = 19.54, G = 36,973) (Fig. 4).

For both insect species used in the experiment, no F1 adult emergence was observed from treated wheat grains during the first four weeks. Consequently, statistical analyses were conducted using data collected between weeks 5 and 10. According to these results, although fewer emergences were observed in *O. surinamensis* compared to *S. oryzae* in weeks 5, 6, and 7, an increase in F1 adult emergence was noted starting from week 8. The first F1 adults were observed in week 5 for both insect species.

In experiments where adults were removed and incubated in wheat after the orientation trial, it was observed that F1 adult emergence was statistically lower for all concentrations compared to the control group. By the end of the 10th week, for both species, F1 adult emergence significantly decreased as the concentrations of GS and ES extracts increased, as well as in the GS + Control and ES + Control extracts. However, when comparing the two methods, it was concluded that F1 adult emergence was higher in the GS + Control and ES + Control extracts compared to the other treatments.

For *S. oryzae*, F1 adult emergence was primarily observed in the GS 62.5 + control and GS extracts, with emergence gradually decreasing as the concentrations of GS + Control and GS extracts increased, with counts of 89 and 77, respectively, and cessation by the 10th week. In the case of ES + Control and ES extracts, emergence decreased as the concentrations increased. When comparing GS + Control, ES + Control, GS, and ES, the emergence was statistically highest in the GS + Control extracts, with a count of 89 at the 10th week. When GS and GS + Control were compared with ES and ES + Control, it was determined that the highest F1 adult emergence occurred from the wheat grains infected in the response trials involving GS and ES + Control (Fig. 4).

In the *O. surinamensis* experiments, the first emergences were observed in the control group during the 5th week, while in the experiments with extracts, the first emergences were noted in the 8th week. When comparing the concentrations of GS, ES, GS + Control, and ES + Control extracts, it was found that emergence in the GS + Control extracts was higher than in all other extracts, consistent with the results for *S. oryzae*. The F1 adult emergences were recorded as 52, 33, 31, and 20 individuals in the GS 62.5 + control, ES + control, GS 62.5, and ES 62.5 extracts, respectively (Fig. 4).

### Comparative phenolic profiles of ES (oven-dried) and GS (shade-dried) aqueous extracts obtained from *Trachystemon orientalis*

The quantitative phenolic composition of the aqueous extracts (ES and GS), previously determined by LC–MS/MS^41^, is summarized in Supplementary Table S1 for comparative purposes. Among the commonly detected compounds, *p*-salicylic acid was the predominant phenolic in both extracts, with significantly higher levels in ES (40,173.50 mg/10^6^ g dm), compared to GS (*p* < 0.05). *o*-Salicylic acid was also significantly enriched in ES (2038.00 mg/10^6^ g dm), whereas GS contained markedly lower concentrations. In contrast, abscisic acid was substantially higher in GS (3346.76 mg/10^6^ g dm) than in ES (*p* < 0.05). Vanillin and *p*-coumaric acid differed significantly between the two extracts, with vanillin being higher in GS and *p*-coumaric acid being higher in ES (*p* < 0.05). Protocatechuic acid also showed a significant difference, with greater accumulation in ES (1052.19 mg/10^6^ g dm). Regarding extract-specific compounds, rosmarinic acid was detected exclusively in ES (956.11 mg/10^6^ g dm), whereas caffeic acid was present only in GS (*p* < 0.05). In contrast, chrysophanol did not differ significantly between ES and GS (Table S1).

## Discussion

The current study presents a novel approach to pest management by investigating the effects of two aqueous extracts derived from *T. orientalis* on two major stored-product pests, *S. oryzae* and *O. surinamensis*. This research marks the first attempt to explore the potential of this specific plant, belonging to the Boraginaceae family, against stored-product pests, thus filling a critical gap in the literature. The orientation assay revealed significant concentration- and time-dependent changes in the distribution of both insect species among treated, control, and neutral compartments. For both pests, GS and ES extracts generally reduced the proportion of insects recorded in treated areas at early time points, suggesting an avoidance response. However, at later time points, a higher proportion of adults was observed in treated compartments for some treatments, indicating a temporal change in behavioral response rather than a consistent repellent or attractive effect. The mechanisms underlying these temporal changes are not addressed by the present experimental design. Such changes may be associated with temporal variation in volatile composition, sensory adaptation, or attenuation of avoidance behavior, and further olfactometric and chemical analyses would be necessary to elucidate these processes. In particular, the GS extract attracted *S. oryzae* adults toward the control group at all concentrations, highlighting its potential as a potent botanical repellent. Our observations align with previous reports on the repellent effects of plant-derived bioactive compounds from other Boraginaceae family members. For instance, Asiry et al.^44^ demonstrated that *H. bacciferum* exhibited moderate repellent activity against *O. surinamensis*, achieving a repellent rate of 55% at a concentration of 500 ppm. Similarly, *Symphytum officinale* L. and *B. officinalis* (Boraginaceae) showed significant antifeedant activity against *Leptinotarsa decemlineata* Say (Coleoptera: Chrysomelidae) and *Spodoptera littoralis* (Boisduval) (Lepidoptera: Noctuidae)^66^. Other studies have highlighted the eco-friendly potential of *Borago officinalis* (Boraginaceae) for controlling pests such as *Spodoptera littoralis*^67^, *Rhynchophorus ferrugineus* (Oliver) (Coleoptera: Curculionidae)^68^, and *Myzus persicae* (Hemiptera: Aphididae)^69^. Crude and ethyl acetate extracts of *Moltkiopsis ciliata* (Forssk 1953) (Boraginaceae: I. M. Johnst.), obtained using the air drying method, exhibited highly toxic effects on *S. oryzae*. However, the overall effect of *M. ciliata* was not statistically significant in terms of repellent effects^43^. These findings provide a strong basis for the significant activity of *T. orientalis* extracts observed in this study, emphasizing its unique chemical profile and the active compounds responsible for its effects.

Phytochemical screening of the GS and ES extracts revealed the presence of several phenolic compounds, including caffeic acid, protocatechuic acid, and salicylic acid, which may have contributed to the observed bioactivity. The biological activity of caffeic acid on insects, which was high in GS extract in this study and widely documented in other studies, has been reported. For example, Zarmakoupi et al.^70^ reported mortality rates of 73% in *Trogoderma granarium*, and 67% in both *Cryptolestes ferrugineus* and *Tribolium confusum* exposed to caffeic acid. Similarly, caffeic acid methyl ester exhibited strong larval inhibitory activity against *Spodoptera litura* and *Achaea janata*^71^. Beyond its direct toxicity, caffeic acid derived from *Urtica dioica* showed repellency against *Aphis gossypii* and *Phenacoccus solenopsis*, with deterrence rates of 46.2% and 43.5%, respectively^72^. Previous studies have indicated that the accumulation of caffeic acid under heavy aphid infestation reinforces cell wall defenses and deters aphid feeding. At the same time, a decline in protocatechuic acid levels suggests a dynamic interplay in plant defensive metabolism^73^.

Protocatechuic acid, particularly found at high levels in the ES extract in our study, may also play an indirect role in insect-plant interactions^74^. Its variation across plant tissues has been associated with the feeding preferences and developmental performance of *Heliothis armigera* and *Spodoptera litura*, potentially influencing host selection^75^. Although protocatechuic acid showed limited direct toxicity, its interaction with salicylic acid and caffeic acid may yield combined effects that enhance repellency and oviposition deterrence.

Beyond phenolic acids, other signaling molecules such as abscisic acid have also been implicated in plant resistance which may have a negative effect on pests. Exogenous abscisic acid application was found to activate systemic defenses in tomato, reducing *Trialeurodes vaporariorum *performance^76^, while Taylor et al.^77^ showed its involvement in mosquito physiology by reducing developmental and reproductive success. These findings suggest that the phenolic and signaling compounds identified in *T. orientalis* extracts may contribute both directly and indirectly to the suppression of stored-product pests, either through toxicity, repellency, or disruption of developmental processes.

The current study also included a group that did not exhibit an orientation response to the plant extracts tested in the three-compartment Petri dish experiment. While increasing neutrality was generally observed in *O. surinamensis* depending on the concentration, this situation was more pronounced against both extracts in *S. oryzae*, and the highest neutrality was observed against the ES extract. This neutral effect could be explained by several factors. The concentration tested could be below the insects’ detection threshold or excessively high, which could lead to a masking or inhibitory effect on their olfactory receptors^78–80^. The general lack of response with increasing concentration in both insects suggests that their sensory systems may have been suppressed by the high concentration. There are very few studies in the literature on this neutral effect or lack of response^81–83^. Avoiding choice may also be a defensive strategy for pests. To clarify this more clearly, future research should optimize extraction methods and concentrations and utilize different experimental designs (e.g., olfactometry).

In contrast, when behavioral differences were detected, the results revealed species-specific preferences. *Oryzaephilus surinamensis* showed a stronger preference toward the control group, particularly when the ES extract was applied, while *S. oryzae* showed a preference toward the control group at all concentrations of the GS extract. These divergent responses may be related to interspecific differences in olfactory sensitivity or ecological adaptation, as stored-product insects rely on different sets of volatiles associated with their host substrates^84,85^. Similar species-dependent variability in responses to plant-derived volatiles has also been documented in other insect groups^86^. From a practical standpoint, these findings emphasize that plant extracts should not be assumed to have uniform behavioral effects across pest species. Instead, they highlight the necessity of species-specific evaluations when considering the use of botanical extracts as behavioral modifiers in integrated pest management programs.

A striking outcome of this study is the significant reduction in F1 adult emergence observed across all treated groups. These findings confirm that the concentrations used in the orientation assay were biologically relevant and sufficient not only to elicit behavioral responses but also to significantly suppress F1 adult emergence, demonstrating that short-term exposure was adequate to evaluate progeny-related effects. The gradual decrease in F1 adult emergence with increasing extract concentrations suggests a concentration-dependent inhibition of oviposition or larval development. Among the treatments, the combinations of GS + Control and ES + Control showed higher hatching rates than the extracts alone, probably due to the attractive effect of untreated grains in the mixed setup. For both *S. oryzae* and *O. surinamensis*, the extracts effectively suppressed larval development, with higher concentrations showing the most pronounced effects. This observation is consistent with studies on Boraginaceae extracts, which exhibited oviposition-inhibiting and larvicidal properties^87–89^. However, the observed differences in F1 adult emergence between the two insect species highlight potential species-specific susceptibilities to *T. orientalis* extracts, a phenomenon that should be explored further.

Our results showed that after 120 h exposure, insects did not consistently move away from ES-treated areas; in some cases, individuals even accumulated there. This indicates that F1 suppression was not exclusively attributable to spatial avoidance but likely reflects sublethal effects impairing reproductive performance. Similar behavioral–progeny associations have been reported in stored-product beetles^90,91^. F1 production declined in a concentration-dependent manner, supporting a direct inhibitory effect on oviposition. Botanical extracts have been shown to suppress oviposition and F1 development even when adults remain in treated environments^92,93^. The partial recovery observed in diluted treatments further supports a dose-responsive mechanism, consistent with findings reported by Yankanchi and Gadache^94^ and Silva et al.^95^.

The 24–120 h exposure window encompasses the oviposition phase and allows cumulative impacts to manifest. Prolonged exposure has been linked to increased reductions in fecundity and progeny in stored-product insects^90–92^. Although ES exhibited stronger orientation effects, both extracts similarly reduced F1 emergence, indicating that oviposition deterrence and sublethal reproductive impacts played a more decisive role than spatial avoidance alone. The originality of this study lies in its pioneering use of *T. orientalis* against stored-product pests. While other Boraginaceae plants have been studied for their pesticidal properties, this is the first investigation targeting the stored-product pests *S. oryzae* and *O. surinamensis*. Long-term studies assessing the persistence and efficacy of these extracts under practical storage conditions are essential to validate their commercial applicability. In conclusion, the results of this study underscore the potential of *T. orientalis* as a natural pest management agent, offering an innovative and sustainable solution to the challenges posed by stored-product pests.

## Conclusion

The findings of this study demonstrate the significant potential of *T. orientalis* extracts as effective agents against stored product pests, specifically *O. surinamensis* and *S. oryzae*. The orientation behaviors of the pests showed that exposure to GS and ES extracts resulted in an initial preference for control areas, which diminished over time as the pests exhibited increased attraction toward treated compartments. This shift indicates that prolonged exposure to the extracts potentially alters pest behavior, supporting their application as attractant or repellent agents under specific conditions. Additionally, the study revealed a concentration-dependent decrease in F1 adult emergence, with higher GS and ES extract concentrations effectively limiting F1 adult emergence in both pest species. The F1 adult emergence was observed earlier in the control groups, while delayed and reduced emergence was noted in extract-treated plots, particularly in the GS + Control and ES + Control treatments. These findings underline the potential of these extracts not only as behavioral modifiers but also as inhibitors of pest population growth. Comparisons with related studies further validate the efficacy of extracts from the Boraginaceae family, which have shown variable but promising toxicological and repellent effects across a range of pest species. The study’s results align with previous findings on the insecticidal, antifeedant, and repellent properties of Boraginaceae extracts, emphasizing their potential as eco-friendly pest management solutions. This research offers a novel perspective on the application of *T. orientalis* extracts, setting a foundation for future exploration into their use in integrated pest management systems. Further studies are recommended to evaluate the long-term stability, field efficacy, and potential synergistic effects of these extracts with other pest control methods. Future research should focus on (i) identifying the volatile and non-volatile compounds responsible for the observed behavioral effects, (ii) evaluating the stability and persistence of these extracts under real storage conditions, (iii) assessing sublethal impacts on oviposition behavior and insect physiology, and (iv) exploring their compatibility with other non-chemical control strategies within integrated pest management programs.

## Supplementary Information

Below is the link to the electronic supplementary material.

Supplementary Material 1

## Data Availability

All data supporting the findings of this study that are not available within the manuscript or supplementary information files can be requested from the authors.
