# Supplementary material for: Biological activity of Trachystemon orientalis extracts against Sitophilus oryzae and Oryzaephilus surinamensis
Source: Sci Rep. 2026 Apr 11;16:16889. doi: 10.1038/s41598-026-47834-7 (PMC13230615; doi:10.1038/s41598-026-47834-7)
Supplement: Supplementary file 1 — Supplementary Material 1 [file 41598_2026_47834_MOESM1_ESM.docx]

**Supplementary Table S1.** Comparative phenolic composition of ES (oven-dried) and GS (shade-dried) aqueous extracts of *Tracystemon orientalis* (Data adapted from Susurluk et al. 2025; LC–MS/MS analysis)

| **Phenolic Compound** | **ES (mg/10⁶ g dm)** | **GS (mg/10⁶ g dm)** | **Statistical difference** |
| --- | --- | --- | --- |
| Chrysophanol | 75.40 ± 2.64 | 77.59 ± 19.27 | Not significant |
| Protocatechuic acid  Caffeic acid | 1052.19 ± 3.73  Not detected | 812.32 ± 17.40  1666.17 ± 1.41 | Significant  Significant |
| *o*-Salicylic acid | 2038.00 ± 33.10 | 391.82 ± 5.96 | Significant |
| *p*-Salicylic acid | 40,173.50 ± 347.62 | 33,701.75 ± 20.37 | Significant |
| Vanillin | 426.44 ± 19.43 | 554.19 ± 10.97 | Significant |
| *p*-Coumaric acid | 924.55 ± 21.30 | 646.81 ± 32.13 | Significant |
| Abscisic acid  Dihydrokaempferol | 180.81 ± 1.40  Not detected | 3346.76 ± 6.58  339.94 ± 10.50 | Significant  Significant |
| Rosmarinic acid | 956.11 ± 23.32 | Not detected | Significant |

Phenolic concentrations are expressed as mg/10⁶ g dry matter and reported as mean ± standard deviation. Means were compared using one-way ANOVA followed by Duncan’s multiple range test (P < 0.05).
